# An asymptomatic child of pre-perforation erosion after transcatheter closure of atrial septal defect

**DOI:** 10.1186/s13019-021-01646-0

**Published:** 2021-09-18

**Authors:** Wen-long Zhang, Fei Liang

**Affiliations:** Department of Cardiovascular Surgery, Shandong Provincial Hospital Affiliated to Shandong Fist Medical University, No. 324, Jingwu Road, Jinan, 250021 China

**Keywords:** Atrial septal occluder, Catheter intervention, Early erosion, Pericardial effusion

## Abstract

**Background:**

Fatal pericardial tamponade caused by aortic or atrial perforation due to erosion of atrial septal occluders has been reported previously, but the timing of erosion is uncertain, and the process is also unclear.

**Case presentation:**

We present a case of a 5-year-old boy with erosion of the aorta and atrium by the occluder not leading to perforation or pericardial tamponade because of early detection and timely surgery. A small amount of pericardial effusion may be the only manifestation of early erosion. This case firstly revealed the early process of device erosion in children.

**Conclusions:**

An absent aortic rim may be a higher risk factor for erosion than oversized device for a child, and it is wise to choose a relatively small occluder or change to surgery. This may be helpful for preventing and treating serious complications caused by erosion of the occluder.

## Background

Transcatheter closure of secundum atrial septal defect (ASD) using the Amplatzer septal occluder (ASO) has been demonstrated to be safe and effective [1]. However, erosion of the device through an atrial wall into the aorta or pericardial space is a rare but serious adverse event that can occur after ASD closure. The absolute risk of erosion after ASO implant has been estimated to range from 0.043 to 0.3% [2]. Until now, the clinical manifestations and mechanisms of early erosion have not been reported.

## Case presentation

A 5-year-old boy of secundum ASD without obvious symptoms was presented at our site. Transoesophageal echocardiography demonstrated a defect size of 15.2 mm × 13.6 mm, but with no rim to the posterior wall of the aorta. The patient underwent transcatheter closure with a 15 mm Amplatzer septal occluder (AGA Company, Sweden) with a trivial residual shunt successfully. Post-procedure echocardiography revealed a well-positioned device without pericardial effusion. The patient was extubated 2 days after admission.

At the patient’s 1 month follow-up visit, echocardiography showed correct device position without residual shunt and pericardial effusion. After one month, echocardiography showed a small amount of pericardial effusion. It was proved to be haemopericardium after the puncture examination under the guidance of ultrasound.

**Fig. 1:**
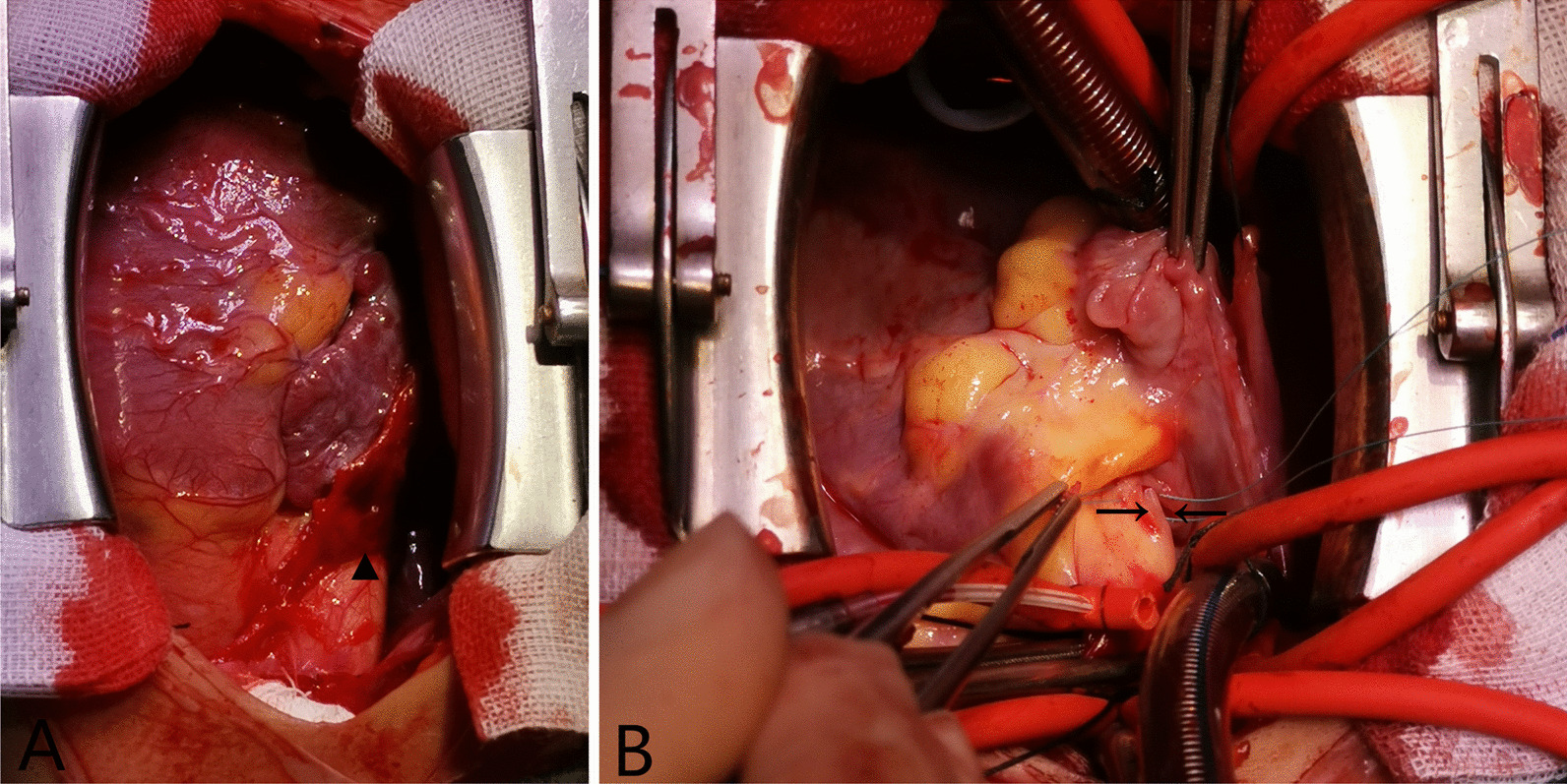
**a** Red thrombosis (the triangle) attached to the anterior wall and root of the aorta. **b** A slit (the rightward arrow) of about 8 mm in length on the outer membrane of the aortic root reaching to the middle layer, and a similarly sized slit (the leftward arrow) on the epicardium of the atrium correspondingly

**Fig. 2:**
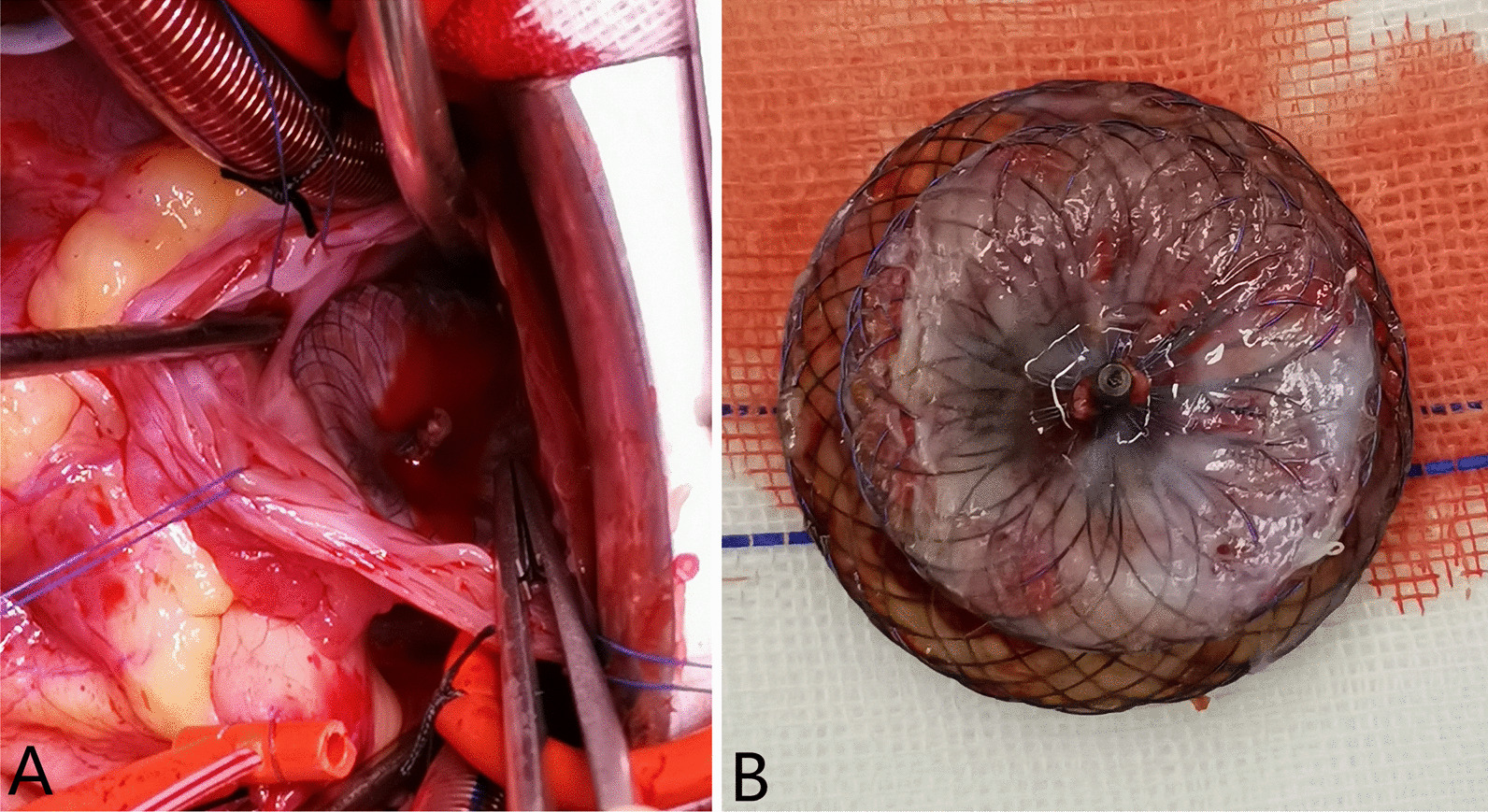
**a** The intact inner wall of right atrium without rupture. **b** Good endothelialization of the atrial septal occluder

After informing the patient's guardians, it was decided to evacuate the occluder by the median sternotomy approach urgently. Intraoperative exploration revealed that there was a red thrombosis (Fig. 1a, the triangle) attached to the anterior wall and root of the aorta; after removing the thrombus, there was a slit of about 8 mm in length (Fig. 1b, the rightward arrow) reaching to the middle layer on the outer membrane of the aortic root, and there was also a similarly sized slit on the epicardium of the atrium correspondingly (Fig. 1b, the leftward arrow). The endothelium of the atrium was intact without rupture (Fig. 2a), and the endothelialization of the occluder was good without thrombus formation (Fig. 2b). The slits on the aortic root and the atrium were repaired using a double-ended needle with gasket respectively, and the ASD was continuous repaired with a polyester patch. The boy was discharged in good condition. No recurrence of pericardial effusion was found during a period of twelve-month follow-up.

## Discussion and conclusions

Cardiac erosion is a potentially fatal complication in catheter closure of ASD and occurs even after a technically adequate procedure. Among erosion patients, a deficient aortic rim (considered deficient if length is < 5 mm; absent if < 1 mm) and oversized device (ASO device should be no larger than 1 to 2 mm above the stop-flow diameter) are at higher risk for cardiac erosion [3]. Usually with a deficient aortic rim, the diameter of the ASD is even larger (> 15 mm in children and > 25 mm in adults), and the left atrium is relatively smaller, so the upper edge of the defect near the aorta is hard to be hugged when the disks of the occluder are released. Some operators will choose oversized occluders. Thomas Herren [4] reported that following percutaneous closure of even large ASDs in children, erosions or late perforations were exceedingly rare**.** As children grow, the ratio of device to atrial septal diameter decreases, whereas this ratio increases over time in adults. But for this case with an absent aortic rim, we didn’t choose an oversized occluder. It suggests that although it is relatively safe to choose an right-sized occluder in a child, erosion may still occur if the aortic rim is absent, and it may occur at an early stage (Within 3 months after surgery). For a child following transcatheter ASD closure, an absent aortic rim may be a higher risk factor for erosion than oversized device. It is wise to choose a relatively small occluder or change to surgery. In addition, a small amount of residual shunt (< 3 mm) during the operation was acceptable, because after a period of follow-up, it was found that the shunts did not increase or even disappeared in most cases.

Concerning the literature, the timing of erosion is variable and risk factors are uncertain [3, 5]. It was guessed that the occluder eroded the atrium firstly, then passed through the atrial wall into the aorta or pericardial space. For this case, the location of the erosion occurred on the adventitia of the aorta and the atrium corresponding to the occluder, but both intima is intact. Following successful ASD occlusion, atrial sizes invariably decrease and thus, make the device to increasingly impinge the atrial wall. A small amount of pericardial effusion may be the only manifestation of early erosion. Early detection and timely surgery can avoid life-threatening complications.

## Data Availability

All data generated or analysed during this study are included in this published article.

## Abbreviations

ASD: Atrial septal defect
ASO: Amplatzer septal occluder
